# Supplementary material for: Awareness of obstetric fistula and its associated factors among reproductive-aged women: Demographic and health survey data from Gambia
Source: PLoS One. 2023 Apr 6;18(4):e0283666. doi: 10.1371/journal.pone.0283666 (PMC10079005; doi:10.1371/journal.pone.0283666)
Supplement: S1 File — (ZIP) [file pone.0283666.s001.zip › Supporting documents/Availability of Data and Materials.docx]

**Availability of Data and Materials**

The dataset is available on the following website: <http://goo.gl/ny8T6X>.
