# Supplementary material for: Awareness of obstetric fistula and its associated factors among reproductive-aged women: Demographic and health survey data from Gambia
Source: PLoS One. 2023 Apr 6;18(4):e0283666. doi: 10.1371/journal.pone.0283666 (PMC10079005; doi:10.1371/journal.pone.0283666)
Supplement: S1 File — (ZIP) [file pone.0283666.s001.zip › Supporting documents/Ethnical approval and consent to participation.docx]

**Ethnical approval and consent to participation**

There was no need for further ethical approval for this study because we used publicly available secondary data. More information regarding the DHS data usage and ethical guidelines can be found at <http://goo.gl/ny8T6X>. All methods were performed in accordance with the relevant guidelines and regulations.
