# Supplementary material for: Awareness of obstetric fistula and its associated factors among reproductive-aged women: Demographic and health survey data from Gambia
Source: PLoS One. 2023 Apr 6;18(4):e0283666. doi: 10.1371/journal.pone.0283666 (PMC10079005; doi:10.1371/journal.pone.0283666)
Supplement: Attachment — Submitted filename: fistula in gambia.pdf [file pone.0283666.s002.pdf]

PLOS ONE

AWARENESS OF OBSTETRIC FISTULA AND ITS ASSOCIATED FACTORS AMONG REPRODUCTIVE-AGED WOMEN: DEMOGRAPHIC AND HEALTH SURVEY DATA FROM GAMBIA

--Manuscript Draft--

**Manuscript Number:**	PONE-D-22-16318
**Article Type:**	Research Article
**Full Title:**	AWARENESS OF OBSTETRIC FISTULA AND ITS ASSOCIATED FACTORS AMONG REPRODUCTIVE-AGED WOMEN: DEMOGRAPHIC AND HEALTH SURVEY DATA FROM GAMBIA
**Short Title:**	Obstetric fistula awareness in Gambia
**Corresponding Author:**	Rabbi Tweneboah, B.A Economics<br>Kwame Nkrumah University of Science and Technology<br>Kumasi, GHANA
**Keywords:**	Obstetric, Fistula, Childbirth complications, Public Health, Gambia
**Abstract:**	Introduction<br>Childbirth complications continue to remain a major problem in various settings but most rampant in underdeveloped nations, including Gambia, where poor living condition is widespread. Obstetric Fistula (OF) has been cited as one of the most common issues experienced by mothers during labor over the years. The study thus focuses on evaluating the level of awareness of this condition among Gambian women of childbearing age.<br>Methods<br>Women's Data from the recent Demographic and Health Survey (DHS) in Gambia was used for the study. A total of 11,864 women of reproductive age, who had completed cases of the variables of interest were used for the analysis. Stata version-16 was used in carrying out the analysis of this study; and Pearson Chi-square test for independence was used to examine the distribution of the awareness of fistula among Gambian women across the explanatory variables. A two model binary logistic regression was fitted to examine the association between the outcome variable and the explanatory variables.<br>Results<br>The study presented that, majority of the Gambian women (87.2%) had no knowledge about Obstetric Fistula, as they indicated to have never heard of the condition. Considering the individual factors, age was seen to be a significant factor in determining the awareness level of Obstetric Fistula among women of childbearing age. As they age, the higher their odds of knowing about the condition. Other factors such as level of education, marital status, pregnancy termination, media exposure, community poverty level, and employment were also discovered to be significant factors in determining a woman's awareness of Obstetric Fistula.<br>Conclusion<br>Considering the low awareness level of Obstetric Fistula among Gambian women, there is therefore the need for the appropriate institutions to increase health educational programmes targeted at creating its awareness, and to provide further in-depth understanding of the condition to the few who already have a fair knowledge about it.
**Order of Authors:**	Rabbi Tweneboah
	Eugene Budu
	Patience Dzigbordi Asiam
	Stephen Aguadze
	Franklin Acheampong
**Additional Information:**	
**Question**	**Response**

Powered by Editorial Manager® and ProduXion Manager® from Aries Systems Corporation

**Financial Disclosure**<br><br>Enter a financial disclosure statement that describes the sources of funding for the work included in this submission. Review the submission guidelines for detailed requirements. View published research articles from *PLOS ONE* for specific examples.<br><br>This statement is required for submission and **will appear in the published article** if the submission is accepted. Please make sure it is accurate.<br><br>**Unfunded studies**<br>Enter: *The author(s) received no specific funding for this work.*<br><br>**Funded studies**<br>Enter a statement with the following details:<br>• Initials of the authors who received each award<br>• Grant numbers awarded to each author<br>• The full name of each funder<br>• URL of each funder website<br>• Did the sponsors or funders play any role in the study design, data collection and analysis, decision to publish, or preparation of the manuscript?<br>• **NO** - Include this sentence at the end of your statement: *The funders had no role in study design, data collection and analysis, decision to publish, or preparation of the manuscript.*<br>• **YES** - Specify the role(s) played.<br><br>* typeset	The authors received no specific funding for this work
**Competing Interests**<br><br>Use the instructions below to enter a competing interest statement for this submission. On behalf of all authors, disclose any competing interests that could be perceived to bias this work—acknowledging all financial support and any other relevant financial or non-financial competing interests.<br><br>This statement is required for submission	The authors have declared no competing interest exist.

and **will appear in the published article** if the submission is accepted. Please make sure it is accurate and that any funding sources listed in your Funding Information later in the submission form are also declared in your Financial Disclosure statement.<br><br>View published research articles from *PLOS ONE* for specific examples.<br><br>**NO authors have competing interests**<br><br>Enter: *The authors have declared that no competing interests exist.*<br><br>**Authors with competing interests**<br><br>Enter competing interest details beginning with this statement:<br><br>*I have read the journal's policy and the authors of this manuscript have the following competing interests: [insert competing interests here]*<br><br>* typeset	
**Ethics Statement**<br><br>Enter an ethics statement for this submission. This statement is required if the study involved:<br><br>• Human participants<br>• Human specimens or tissue<br>• Vertebrate animals or cephalopods<br>• Vertebrate embryos or tissues<br>• Field research<br><br>Write "N/A" if the submission does not require an ethics statement.<br><br>General guidance is provided below. Consult the submission guidelines for detailed instructions. **Make sure that all information entered here is included in the Methods section of the manuscript.**	N/A

**Format for specific study types**<br><br>**Human Subject Research (involving human participants and/or tissue)**<br>• Give the name of the institutional review board or ethics committee that approved the study<br>• Include the approval number and/or a statement indicating approval of this research<br>• Indicate the form of consent obtained (written/oral) or the reason that consent was not obtained (e.g. the data were analyzed anonymously)<br><br>**Animal Research (involving vertebrate animals, embryos or tissues)**<br>• Provide the name of the Institutional Animal Care and Use Committee (IACUC) or other relevant ethics board that reviewed the study protocol, and indicate whether they approved this research or granted a formal waiver of ethical approval<br>• Include an approval number if one was obtained<br>• If the study involved *non-human primates*, add *additional details* about animal welfare and steps taken to ameliorate suffering<br>• If anesthesia, euthanasia, or any kind of animal sacrifice is part of the study, include briefly which substances and/or methods were applied<br><br>**Field Research**<br><br>Include the following details if this study involves the collection of plant, animal, or other materials from a natural setting:<br>• Field permit number<br>• Name of the institution or relevant body that granted permission	
**Data Availability**<br><br>Authors are required to make all data underlying the findings described fully available, without restriction, and from the time of publication. PLOS allows rare exceptions to address legal and ethical concerns. See the PLOS Data Policy and FAQ for detailed information.	Yes - all data are fully available without restriction

A Data Availability Statement describing where the data can be found is required at submission. Your answers to this question constitute the Data Availability Statement and **will be published in the article**, if accepted.<br><br>**Important:** Stating 'data available on request from the author' is not sufficient. If your data are only available upon request, select 'No' for the first question and explain your exceptional situation in the text box.<br><br>Do the authors confirm that all data underlying the findings described in their manuscript are fully available without restriction?	
**Describe where the data may be found in full sentences. If you are copying our sample text, replace any instances of XXX with the appropriate details.**<br><br>• If the data are **held or will be held in a public repository**, include URLs, accession numbers or DOIs. If this information will only be available after acceptance, indicate this by ticking the box below. For example: *All XXX files are available from the XXX database (accession number(s) XXX, XXX.)*.<br>• If the data are all contained **within the manuscript and/or Supporting Information files**, enter the following: *All relevant data are within the manuscript and its Supporting Information files.*<br>• If neither of these applies but you are able to provide **details of access elsewhere**, with or without limitations, please do so. For example:<br><br>*Data cannot be shared publicly because of [XXX]. Data are available from the XXX Institutional Data Access / Ethics Committee (contact via XXX) for researchers who meet the criteria for access to confidential data.*<br><br>*The data underlying the results presented in the study are available from (include the name of the third party*	The data underlying the results presented in the study are available from Measuredhs. www.measuredhs.com

and contact information or URL).<br>• This text is appropriate if the data are owned by a third party and authors do not have permission to share the data.<br><br>* typeset	
Additional data availability information:	

Powered by Editorial Manager® and ProduXion Manager® from Aries Systems Corporation

Manuscript

Click here to access/download;Manuscript;Manuscript.docx

AWARENESS OF OBSTETRIC FISTULA AND ITS ASSOCIATED FACTORS AMONG REPRODUCTIVE-AGED WOMEN: DEMOGRAPHIC AND HEALTH SURVEY DATA FROM GAMBIA

Eugene Budu[1], Rabbi Tweneboah[2], Patience Dzigborbi Asiam[3], Stephen Aguadze[4], Franklin Acheampong[1]

[1]Research Unit, Korle Bu Teaching Hospital, P.O. Box77, Accra, Ghana

[2]Department of Economics, Kwame Nkrumah University of Science and Technology, Ghana

[3]Department of Statistics, Kwame Nkrumah University of Science and Technology, Ghana

[4]Korle Bu Teaching Hospital, P.O. Box77, Accra, Ghana

***Corresponding Author**

Tweneboah Rabbi

Email addresses:

EB: budueugene@gmail.com

RT: antwirabbi56@gmail.com

PDA: asiam.patience@gmail.com

SA: Stephaguadze@gmail.com

FA: acheampongkbth@gmail.com

Abstract

Introduction

Childbirth complications continue to remain a major problem in various settings but most rampant in underdeveloped nations, including Gambia, where poor living condition is widespread. Obstetric Fistula (OF) has been cited as one of the most common issues experienced by mothers during labor over the years. The study thus focuses on evaluating the level of awareness of this condition among Gambian women of childbearing age.

Methods

Women's Data from the recent Demographic and Health Survey (DHS) in Gambia was used for the study. A total of 11,864 women of reproductive age, who had completed cases of the variables of interest were used for the analysis. Stata version-16 was used in carrying out the analysis of this study; and Pearson Chi-square test for independence was used to examine the distribution of the awareness of fistula among Gambian women across the explanatory variables. A two model binary logistic regression was fitted to examine the association between the outcome variable and the explanatory variables.

Results

The study presented that, majority of the Gambian women (87.2%) had no knowledge about Obstetric Fistula, as they indicated to have never heard of the condition. Considering the individual factors, age was seen to be a significant factor in determining the awareness level of Obstetric Fistula among women of childbearing age. As they age, the higher their odds of

knowing about the condition. Other factors such as level of education, marital status, pregnancy termination, media exposure, community poverty level, and employment were also discovered to be significant factors in determining a woman's awareness of Obstetric Fistula.

Conclusion

Considering the low awareness level of Obstetric Fistula among Gambian women, there is therefore the need for the appropriate institutions to increase health educational programmes targeted at creating its awareness, and to provide further in-depth understanding of the condition to the few who already have a fair knowledge about it.

Keywords: Obstetric, Fistula, Childbirth complications, Gambia

Introduction

Obstetric fistula is one of the most devastating and catastrophic childbirth injuries. It is characterized by a hole which occurs between the birth canal and the bladder, where the rectum is sometimes included as well. This is caused by delayed, complicated labour without access to early, high-quality medical attention. It causes women and girls to leak fluids (i.e. faeces, urine) or both, and frequently leads to persistent medical issues, hopelessness, social isolation, and hardship (1). Fistula affects an estimated half a million women and girls in Sub-Saharan Africa, Asia, the Arab States area, and Latin America and the Caribbean, with new instances emerging every year. Yet fistula is pretty much fully controllable (1,2).

Its recurrence is a clear indication of vast discrepancies, a symptom of global inequality, and proof that health and wellbeing institutions are failing to safeguard the administration of human rights of the economically marginalized women and girls. Precise obstetric fistula prevalence numbers (globally and nationally) are unknown owing to faulty reporting, under reporting, and embarrassment, which prevents women from making complaints about fistula. However, an estimated 50,000 to 100,000 women suffer fistula each year, with around 2 million women already living with the condition, which is a burden in nearly 60 nations (3). Based on data from two African countries, including The Gambia, the most recent community-based prevalence estimate is 160 (95 percent CI 116–210) obstetric fistulas per 1000 women of reproductive age (4).There are several nations in South Asia, notably Bangladesh, and in sub-Saharan Africa, such as Sudan, Ethiopia, Chad, Ghana, and Nigeria, where fistula incidence is estimated to be prominent (5).

A variety of studies have discovered that women's educational level, age, history of pregnancy, distance to the nearest health facility, and awareness of obstetrics problems are the primary elements linked with women's awareness of obstetrics fistula (3,6). While prolonged labour and a lack of timely accessibility to emergency obstetric treatment are the most common leading antecedents of obstetric fistula in underdeveloped countries, widespread poverty is frequently a fundamental factor. According to studies, fistula sufferers tend to reside in rural places and are more disadvantaged (7,8).

Given the negative implications of fistula on the health of women and girls, management and rehabilitation are major public health problems. It is projected that if all affected women received treatment, given the current pace of surgical therapy of fistulas, it will take nearly 55 years to repair all existing sufferers, let alone tackle the new cases that develop each year (9). Surgical intervention to correct the fistula is followed by therapy, which involves extending and moving limbs that have ceased to function as a result of genital tract and sciatica nerve injury. Lower limb paralysis, foot drop, and limb contracture require physiotherapy to be treated (10). Psychological and emotional therapy, skill improvement, and outreach to identify women with perforations and transfer them to distant treatment clinics help solidify treatment activities (11). Although fistula repairs have a success rate of up to 90%, many women are unaware that treatment for their illness is actually available (5,12) and a greater number of women are not even aware of the obstetric fistula condition. In response, Gambia launched a 2017-2021 project with the goal of funding at least 150 fistula repair procedures in The Gambia (13). As of 2021, Gambia, in collaboration with other institutions, were able to support 19 repair procedures, giving hope to women suffering from this illness. It is

undeniable that the first step in resolving any health issue is being aware of its existence and identifying it. However, there is a paucity of evidence on the awareness of women in Gambia on OF. The study therefore aims at investigating the awareness of obstetric fistula and its associated factors among Gambian women of reproductive age.

Methods

Data source

Data from the recent Demographic and Health Survey (DHS) in Gambia was used for the study. Specifically, the study used the women's recode file also known as the Individual Recode (IR). DHS is a comparable nationally representative survey undertaken regularly in over 90 countries, enhancing global understanding of developing country health and demographic trends (14). The DHS Program's major goal is to improve demographic, health, and nutrition data collection, analysis, and distribution, as well as to make these data more useful for planning, policymaking, and program management (14).

Study design and sampling procedure

A descriptive cross-sectional design was employed for the survey. Validated and pretested structured questionnaires were used to collect data from the respondents on health and social issues such as maternal health service utilization and women empowerment and sociodemographic characteristics (14,15). The survey was conducted using a two-stage cluster sampling technique. To begin, a stratified sample of enumeration areas (EAs) is chosen using probability proportional to size (PPS): a sample of a preset number of EAs is chosen independently in each stratum using probability proportional to the EA's measure of size. A listing technique is used in the designated EAs to ensure that all dwellings/households are listed. Second, households in the selected EAs are selected using equal probability systematic sampling. A detailed procedure for sampling has been described elsewhere (16). We included 11,864 women with complete cases of variables of interest in the study. We also adopted the

STROBE (Strengthening the Reporting of Observational Studies in Epidemiology) guidelines in drafting this manuscript (17). The dataset is freely available to download at https://dhsprogram.com/methodology/survey/survey-display-555.cfm.

Variables

Outcome variable

The outcome variable of this study was women's awareness of obstetric fistula. The variable measures the extent to which women are aware of obstetric fistula. This variable was derived from the question "have you heard about fistula?" Responses to this question was categorised into "no" and "yes". The variable was dichotomised into 1= "ever heard of fistula" and 0= "never heard of fistula". Studies that used the DHS dataset employed similar coding (2,18,19).

Explanatory variables

The explanatory variables considered in this study were selected based on their association with awareness of fistula from literature (2,18,19) and also their availability in the DHS dataset. A total of seventeen (17) variables were included in the study. These variables can be grouped as individual and contextual factors. The individual factors considered were mother's age, educational level, marital status, religion, employment status, parity, wealth index, frequency of reading newspaper, frequency of listening to radio, frequency of watching television, sexual activity, pregnancy status and pregnancy termination. The contextual factors included were type of place of residence, region, community literacy level and community poverty level. The categories of each of the variables are shown in Table 2.

Statistical analyses

Stata version 16.0 was used to carry out the analysis in four steps. At the first stage, a graphical chart was used to summarize the results of the proportion of awareness of fistula among women in Gambia. The Pearson chi-square test of independence was adopted to examine the distribution of the awareness of fistula among women across the explanatory variables. A multi-collinearity test using the variance inflation factor (VIF) was conducted to examine the collinearity among the variables. The results indicated that the minimum, maximum, and mean VIFs were 1.04, 6.70, and 2.26 respectively; hence, there was no evidence of collinearity among the variables included in the regression analysis. Finally, a two model binary logistic regression was fitted to examine the association between the outcome variable and the explanatory variables. In the first model (Model I), there was a bivariate binary logistic regression where each of the independent variables was fitted. In the second model, Model II, which is the complete model, a multivariate binary logistic regression was fitted. Odds Ratio of 95 percent confidence intervals (95% CIs) was used to present the findings of the regression analysis. To account for disproportionate sampling and non-response, the "svyset" command was used, and weighting was done to account for the intricate nature of DHS data.

Ethical Approval

The survey reported that ethical approval was granted by the Institutional Review Board of ICF International and Ethical Review Committee of Gambia Health Service (20). We further obtained permission from the DHS Program for use of this data for the study. The data can be accessed from their website (www.measuredhs.com).

Results

Prevalence of Fistula awareness among women in Gambia

Figure 1 provides a graphical representation of fistula awareness level among Gambian women considered for the study. Eighty-seven percent (87.2%) forming the majority of women in Gambia are not aware of fistula. Only 12.8% of women considered for the study had knowledge on fistula.

[Insert Fig 1]

Table 1 summarizes the proportion of fistula awareness in Gambia based on the socio demographic characteristics of the study. Women aged 45-49 had the highest proportion of fistula awareness (20.1%), while women aged 15-19 had the lowest proportion (6.1%). Fistula awareness among women who had never married was only (8.5%) whereas the awareness level among married women was 14.9%. Women who had four or more birth had the greatest proportion of fistula awareness (16.1%) while women with no births formed the least proportion (9.5%). The awareness level among working women was 15.5% and that of women who are not working was 8.9%. Islamic women had the least proportion of fistula (12.6%) whilst the greatest proportion was recorded among women with other religion (41.3%).

Women who read newspaper, listened to radio and watched TV for at least once a week had the highest proportion of fistula awareness (20.2%, 14.9% and 14%) respectively. About two in

ten (17.1%) of women with the highest wealth index were aware of fistula. Highly educated women had the highest proportion of fistula awareness (28.5%). Only 8.4% of women who had never had sex were aware of fistula whilst women who have had sex before reported 14.6% fistula awareness. Fewer women (12.7%) who are not pregnant were aware of fistula while 14.5% pregnant women were aware of fistula. Also, women who had terminated pregnancy before had a higher fistula awareness (17.6%) whereas women who had never terminated pregnancy before had a lower fistula awareness (11.9%).

In terms of sub region, women in Juntaur recorded the highest proportion of fistula awareness (16.6%) whereas women in Janjanbureh had the least fistula awareness (7.7%). Regarding community literacy and poverty level, women in high literacy and high poverty communities had the greatest fistula awareness (14.3% and 15%) respectively. The chi-square test analysis indicated statistically substantial association between all the explanatory variables and fistula awareness except currently pregnant and type of place of residence (see, Table 1).

[Insert table 1]

The outcome of the Binary Logistic Regression has been presented in Table 2. At the bivariate level (Model 1), Compared to women in 15-19 age bracket, the highest odds of fistula awareness were women in their 45 to 49 years (AOR=3.94, CI=3.11-4.99), followed by 30-34 aged women (AOR=2.92, CI=2.38-3.58). Women aged 20-24 had less odds of fistula awareness

compared to those aged 15-19. After adjusting for the covariates in model II, women aged 45-49 years were twice more likely to be aware of fistula than women aged 15-19 (COR=2.19, CI=1.55-3.10). Also women aged 40-44 had higher odds of fistula awareness compared to women aged 15-19 (COR=1.71, CI=1.23-2.38).

With married women as the reference, women that were not married were 46% less likely to be aware of fistula (AOR=0.54, CI=0.47-0.62). Similar results were obtained after adjusting for the other socio demographic factors, unmarried women had less odds of fistula awareness (COR=0.70, CI=0.53-0.92). Compared to women with no birth, women with four or more births had the highest odds of fistula awareness (AOR=1.92, CI=1.68-2.20), followed by women with three births (AOR=1.69, CI=0.79-1.39). Women with one birth are 1.28 times more likely to be aware of fistula than women with no birth (AOR=1.28, CI=1.06-1.55); then women that are working are more likely to be aware of fistula than women that are not working (AOR=1.88, CI=1.66-2.12). Again, Christian women were 1.60 times more likely to be aware of fistula than Islamic women.

Women that listen to radio and read newspapers at least once a week had higher odds of fistula awareness than women that do not listen to radio or read newspapers (respectively; AOR= 1.40, CI=1.23-2.83 & AOR=2.15, CI=1.64-2.83). After adjusting for the covariates, women who listen to radio at least once a week and read newspapers less than once a week had a higher odds of fistula awareness than women who do not listen to radio or read newspapers. Compared to the poorest women in Gambia, women with the wealthiest background were 1.46 times more likely to be aware of fistula (COR=1.46, CI=1.25-1.70). Women with higher education are approximately 3 times more likely to be aware of fistula than women with no

education (AOR=3.27, CI=2.70-3.96). Adjusting for the covariate effects, women with higher education had the highest odds of fistula awareness (COR=3.54, CI=2.74-4.59), followed by women with secondary education (COR=1.38, CI=1.17-1.62), compared to women with no education. Also, women who have never had sex were 47% less likely to be aware of fistula than women who have had sex. Women who have had abortion were more likely to be aware of fistula, compared to women who had never terminated pregnancy. Regarding sub region, women from Janjanbureh had the least odds of fistula awareness (AOR=0.55, CI=0.42-0.72), followed by women in Kerewan (AOR=0.65, CI=0.51-0.83), then by Basse women (AOR= 0.69, CI=0.55-0.87), compared to women in Banjul region. Adjusting for the effects of other factors in model II, women in Juntaur region had 1.92 (CI=1.39-2.65) and those in Mansakonko had 1.60 (CI=1.17-2.18). With women from low literacy community and women from low poverty community as the reference, women from high literacy community had greater odds of fistula awareness (AOR=1.26, CI=1.10-1.43) and women from high poverty community had 1.269 (1.15-1.45).

[Insert table 2]

Discussion

Mitigating obstetric fistula (OF) is critical to the attainment of SDG target 3.1. The study looked at the level of awareness obstetric fistula and the factors that influence it in Gambian women of reproductive age. Overall, the prevalence of OBF awareness in Gambia was 12.9%. This result

was much lower than studies in Nigeria and Tanzania (57.8% and 60.1%) (21,22) respectively. A plausible justification for this finding can be attributed to the differences in study period, design, and sample size. Nevertheless, the finding suggest that fistula awareness is very low in Gambia. This may have a detrimental effect on African countries' quest to mitigate obstetric fistula as women in Gambia are more likely to stay home rather than seek medical intervention (23).

According to the findings, age, marital status, employment position, media exposure, and educational level were all significant socio-demographic factors associated with fistula awareness. The likelihood of being aware of fistula increases with age (e.g. women in 45-49 age bracket were twice likely to be aware of fistula compared to those in 15-19). That is, older women are more likely to be aware of fistula than younger women. This result is in line with prior research from Ethiopia (24) and Uganda (25). The fundamental assumption behind this assertion is that, the older a woman gets, the more experience she would have with birth and its concomitant complications. Our study also revealed that unmarried women in Gambia are less likely to be aware of fistula compared to married women. This is largely due to the influence of the husbands of the married women. Married women, due to the influence of their partners are more likely to seek healthcare services such as obstetric counselling which creates an avenue of increasing awareness among them. There is also the possibility of knowledge transfer from husband to wife. The decision making power of a Gambian woman to visit health institution during labour is very low, the authority is largely made by their husbands (26).

Compared to women who were not exposed to mass media (i.e. reading newspapers and listening to radio), women who were exposed to mass media were more likely to be aware of obstetric fistula. The findings of prior investigations in Nigeria (18) and Ethiopia (27) complement the findings of this study. The finding is probably due to the fact that the media is a vital conduit for conveying information, such as information regarding OF, its signs and symptoms, and information about where to get treatment. As enshrined in (6), most women who are aware of fistula got the information from the media.

Higher level of education among women of reproductive age directly translate to higher health literacy. Education level is a significant predictor of women's awareness of fistula in Gambia. It has been established in several studies (28) that education gradient positively associate with better health behaviours (such as seeking obstetric counselling) and improved health status (fistula awareness). Several other studies (6,29,30) had similar findings that women with higher education are more likely to have improved health status (aware of fistula) compared to those without formal education. Formal education empowers women to make better healthcare decision such as attending health education forums and alter habits that are detrimental to ones' health.

As expected, there is a strong association between fistula awareness and pregnancy termination. Women who had history or were exposed to pregnancy termination were 1.21 times more likely to have better awareness of fistula than those who had not. The finding could be rationalized by the fact that pursuing healthcare services such as pregnancy termination services presents an avenue for women to be exposed to health education and

promoting messages, perhaps raising their awareness of fistula. Similar finding was reported by Aleminew et al. (23) and Asefa et al. (27).

Compared to Banjul, which is a major urban concentration in Gambia, women in these rural towns (Juntaur and Mansakonko) are 1.92 and 1.60 times more likely to be aware of fistula. The high prevalence of obstetric fistula in rural areas in Gambia (31) could explain why awareness of fistula is high also in these towns. However, the finding deviates from the findings of several similar studies which posits that awareness is rather high in urban areas (6,26).

Strength and weakness

By far, our literature review shows that this is the first study to use nationally representative datasets from Gambia to investigate the prevalence of and factors associated with obstetric fistula awareness. This is a significant addition to the existing literature. However, there are some downsides to consider. The DHS does not break down the question to assess which type of fistula women are aware of (e.g., vesicovaginal fistula, urethrovaginal fistula, or rectovaginal fistula). In the future, this disaggregation could be considered for the DHS dataset. Also, the cross-sectional study design does not allow us draw direct causal inference to the factors associated with obstetric fistula awareness.

Conclusion

The present study sought to assess the magnitude and factors associated with fistula awareness in Gambia. We conclude that obstetric fistula awareness is very low in Gambia. As such, immediate remediating action is needed to raise women's awareness about OF. It is

evident from the study that age, level of education, marital status, pregnancy termination, media exposure, community poverty level, and employment status were significant factors associated with OF awareness. To raise women's awareness of fistula, there is the need for public health interventions to consciously raise community literacy rate, increase access to mass media platforms and invest intensively in formal education for women.

Acknowledgement

We thank measuredhs for giving us access to the dataset

Availability of Data and Materials

The dataset is available on the following website: http://goo.gl/ny8T6X.

Authors' Contributions

EB and RT developed the study concept and performed the data analysis. FA, PDA, and SA drafted and revised the manuscript critically for its intellectual content. All authors read and approved the final manuscript.

Funding

The authors received no funding

Declaration

Ethnical approval and consent to participation

There was no need for further ethical approval for this study because we used publicly available secondary data. More information regarding the DHS data usage and ethical guidelines can be found at http://goo.gl/ny8T6X. All methods were performed in accordance with the relevant guidelines and regulations.

Competing interests

The authors declare no competing interests.

Consent for publication

Not applicable.

Reference

1. Sherfi HA, Khogali M, Bedri NM, Blystad A, Hauk H. Experiences of living with Obstetric Fistula (OF) at Khartoum: Case study women attending Abbo's Centre for Fistula and Urogynecology in Khartoum Teaching Hospital. Ahfad J. 2021;38(1).

2. Balcha WF, Nigussie AA, Beyene FY, Tesfu AA. Awareness and Its associated factors of obstetrics fistula among antenatal care attendees in Injibara Town Health Institutions, Awi Zone, North West, Ethiopia, 2019. J Pregnancy. 2020;2020.

3. Rundasa DN, Wolde TF, Ayana KB, Worke AF. Awareness of obstetric fistula and associated factors among women in reproductive age group attending public hospitals in southwest Ethiopia, 2021. Reprod Health. 2021;18(1):1–7.

4. Adler AJ, Ronsmans C, Calvert C, Filippi V. Estimating the prevalence of obstetric fistula: a systematic review and meta-analysis. BMC Pregnancy Childbirth. 2013;13(1):1–14.

5. UNFPA E. Obstetric fistula needs assessment report: Findings from nine African countries. New York UNFPA EngenderHealth. 2003;

6. Banke-Thomas AO, Kouraogo SF, Siribie A, Taddese HB, Mueller JE. Knowledge of obstetric fistula prevention amongst young women in urban and rural Burkina Faso: a cross-sectional study. PLoS One. 2013;8(12):e85921.

7. Treuthart MP. No Woman, No Cry-Ending the War on Women Worldwide and the International VIolence against Women Act (I-VAWA). BU Int'l LJ. 2015;33:73.

8. Hinrichsen D, Richey C, Robey B, Muller F. Obstetric fistula: Ending the silence, easing the suffering. Info Reports. 2004;2.

9. Ahmed S, Tunçalp Ö. Burden of obstetric fistula: from measurement to action. Lancet Glob Heal. 2015;3(5):e243–4.

10. Hilton P. Vesico-vaginal fistulas in developing countries. Int J Gynecol Obstet. 2003;82(3):285–95.

11. Miller S, Lester F, Webster M, Cowan B. Obstetric fistula: a preventable tragedy. J Midwifery Womens Health. 2005;50(4):286–94.

12. Ciriglo J, Larson D, Shirley K, Swain D. Ethiopia: Hamlin Addis Ababa Fistula Hospital.

13. Mocumbi S, Hanson C, Högberg U, Boene H, von Dadelszen P, Bergström A, et al. Obstetric fistulae in southern Mozambique: incidence, obstetric characteristics and treatment. Reprod Health. 2017;14(1):1–10.

14. Croft TN, Marshall AMJ, Allen CK, Arnold F, Assaf S, Balian S. Guide to DHS statistics. Rockv ICF. 2018;645.

15. Corsi DJ, Neuman M, Finlay JE, Subramanian S V. Demographic and health surveys: a profile. Int J Epidemiol. 2012;41(6):1602–13.

16. Health UM of, (Firm) ICFI. Uganda AIDS indicator survey 2011. ICF International; 2012.

17. Von Elm E, Altman DG, Egger M, Pocock SJ, Gøtzsche PC, Vandenbroucke JP, et al. The Strengthening the Reporting of Observational Studies in Epidemiology (STROBE) Statement: guidelines for reporting observational studies. Int J Surg. 2014;12(12):1495–

9.

18. Morhason-Bello IO, Kareem YO, Abdus-Salam RA, Bello OO, Lawal OO, Akinlusi FM, et al. Factors associated with the awareness of vaginal fistula among women of reproductive age: findings from the 2018 Nigerian demographic health cross-sectional survey. BMJ Open. 2020;10(11):e040078.

19. Kasamba N, Kaye DK, Mbalinda SN. Community awareness about risk factors, presentation and prevention and obstetric fistula in Nabitovu village, Iganga district, Uganda. BMC Pregnancy Childbirth. 2013;13(1):1–10.

20. Gambia - Demographic and Health Survey 2019-2020 [Internet]. [cited 2022 Apr 3]. Available from: https://microdata.worldbank.org/index.php/catalog/3980

21. Ezeonu PO, Ekwedigwe KC, Isikhuemen ME, Eliboh MO, Onoh RC, Lawani LO, et al. Awareness of obstetric vesicovaginal fistula among pregnant women in a rural hospital. Adv Reprod Sci. 2017;5(3):39–46.

22. Kazaura MR, Kamazima RS, Mangi EJ. Perceived causes of obstetric fistulae from rural southern Tanzania. Afr Health Sci. 2011;11(3).

23. Aleminew W, Mulat B, Shitu K. Awareness of obstetric fistula and its associated factors among reproductive-age women in Ethiopia: a multilevel analysis of Ethiopian Demographic and Health Survey data: a cross-sectional study. BMJ Open. 2021;11(12):e053221.

24. Rundasa DN, Fekede T, Ayana KB, Worke AF. Awareness of obstetric fistula and

associated factors among women in reproductive age group attending public hospitals in southwest. Reprod Health [Internet]. 2021;1–7. Available from: https://doi.org/10.1186/s12978-021-01228-2

25. den Hollander GC, Janszen EWM. Obstetric fistulas in Uganda: scoping review using a determinant of health approach to provide a framework for health policy improvement. BMC Pregnancy Childbirth. 2020;20(1):1–8.

26. Montez D. Family Planning and Maternal Health in Tanzania: Women Demand for More Information. Africa Dev Res Br Washingt DC Available Available online http//www audiencescapes org/sites/default/files/AudienceScapes-ResearchBriefs-Tanzania-FP-MCH-Montez pdf. 2011;

27. Asefa Z, Amenu D, Berhe A. Awareness of obstetric fistula and its associated factors among reproductive-age group women in bench Sheko zone, Southwest, Ethiopia. community based cross-sectional study. J Women's Heal Care. 2020;9:509.

28. Cutler DM, Lleras-Muney A. Education and health: evaluating theories and evidence. National bureau of economic research Cambridge, Mass., USA; 2006.

29. Gurung S, Panthi V, Kachapati A. Awareness regarding vesicovaginal fistula among women residing in a Municipality of Kapilvastu. J Univers Coll Med Sci. 2018;6(2):46–50.

30. Zhao Q, Kulane A, Gao Y, Xu B. Knowledge and attitude on maternal health care among rural-to-urban migrant women in Shanghai, China. BMC Womens Health.

2009;9(1):1–8.

31. Salami K. Obstetric fistula situation in Gambia.

Tables

Table 1: Distribution of fistula awareness among women in Gambia (N=11864)

Variables	Weighted N	Weighted %	Fistula awareness	P-value
**Age**				<0.001
15-19	2632	22.2	6.1	
20-24	2181	18.4	10.7	
25-29	2248	18.9	14.1	
30-34	1619	13.6	17.2	
35-39	1437	12.1	16.3	
40-44	1028	8.7	15.6	
45-49	718	6.1	20.1	
**Marital status**				<0.001
Not married	3704	31.2	8.5	
Married	7500	63.2	14.9	
Cohabiting	25	0.2	12.9	
Widowed	182	1.5	14.3	
Divorced	453	3.8	13.7	
**Parity**				<0.001
No birth	4321	36.4	9.5	
One birth	1457	12.3	11.4	
Two births	1239	10.5	14.4	
Three births	1205	10.1	15.4	
Four or more births	3641	30.7	16.1	
**Employment status**				<0.001
Not working	4752	40.0	8.9	
Working	7112	60.0	15.5	
**Religion**				0.005
Islam	11442	96.4	12.6	
Christianity	418	3.5	18.6	
Other	4	0.1	41.3	
**Frequency of reading newspaper**				<0.001
Not at all	10124	85.3	12.0	
Less than once a week	1311	11.1	16.7	

At least once a week	429	3.6	20.2	
**Frequency of listening radio**				<0.001
Not at all	2693	22.7	10.8	
Less than once a week	4692	39.5	12.1	
At least once a week	4479	37.8	14.9	
**Frequency of watching TV**				0.047
Not at all	2112	17.8	11.5	
Less than once a week	3144	26.5	11.4	
At least once a week	6608	55.7	14.0	
**Wealth index**				<0.001
Poorest	1998	16.9	11.5	
Poorer	2135	18.0	11.0	
Middle	2292	19.3	11.6	
Richer	2591	21.8	11.8	
Richest	2848	24.0	17.1	
**Level of education**				<0.001
No education	4119	34.7	11.8	
Primary	1854	15.6	12.2	
Secondary	5020	42.3	11.2	
Higher	871	7.4	28.5	
**Sexual activity**				<0.001
Never had sex	3397	28.6	8.4	
Ever had sex	8467	71.4	14.6	
**Currently pregnant**				0.873
Not pregnant	10984	92.6	12.7	
Pregnant	880	7.4	14.5	
**Ever terminated a pregnancy**				<0.001
No	9876	83.2	11.9	
Yes	1988	16.8	17.6	
**Type of place of residence**				0.775
Urban	8746	73.7	13.1	
Rural	3118	26.3	12.3	

**Region**				<0.001
Banjul	162	1.4	14.8	
Kanifing	2589	21.8	14.5	
Brikama	5299	44.7	12.9	
Mansakonko	431	3.6	15.7	
Kerewan	1128	9.5	10.6	
Juntaur	523	4.4	16.6	
Janjanbureh	595	5.0	7.7	
Basse	1136	9.6	10.8	
**Community literacy level**				0.001
Low	2163	18.2	10.3	
Medium	4271	36.0	12.3	
High	5430	45.8	14.3	
**Community poverty level**				<0.001
Low	4379	36.9	11.7	
Moderate	1833	15.4	9.1	
High	5652	47.6	15.0	

Table 2: Association between explanatory variables and fistula awareness among women in Gambia.

Variable	Model I<br>AOR (95% CI)	Model II<br>COR (95% CI)
**Age**		
15-19	Reference (1.0)	Reference (1.0)
20-24	1.81*** (1.47-2.23)	1.35* (1.07-1.70)
25-29	2.38*** (1.96-2.90)	1.55*** (1.20-2.02)
30-34	2.92*** (2.38-3.58)	1.70*** (1.26-2.28)
35-39	2.88*** (2.34-3.55)	1.68** (1.23-2.29)
40-44	2.89*** (2.30-3.62)	1.71** (1.23-2.38)
45-49	3.94*** (3.11-4.99)	2.19*** (1.55-3.10)
**Marital status**		
Not married	0.54*** (0.47-0.62)	0.70* (0.53-0.92)
Married	Reference (1.0)	Reference (1.0)
Cohabiting	0.84 (0.19-3.68)	0.65 (0.15-2.84)
Widowed	0.99 (0.62-1.53)	0.83 (0.53-1.32)
Divorced	1.11 (0.84-1.46)	0.86 (0.64-1.17)
**Parity**		
No birth	Reference (1.0)	Reference (1.0)
One birth	1.28* (1.06-1.55)	0.96 (0.73-1.24)
Two births	1.40*** (1.15-1.71)	0.91 (0.70-1.20)
Three births	1.69*** (1.40-2.05)	1.05 (0.79-1.39)
Four or more births	1.92*** (1.68-2.20)	1.24 (0.94-1.61)
**Employment status**		
Not working	Reference (1.0)	Reference (1.0)
Working	1.88*** (1.66-2.12)	1.43*** (1.25-1.64)
**Religion**		
Islam	Reference (1.0)	Reference (1.0)
Christianity	1.60*** (1.18-2.17)	1.22 (0.86-1.71)
Other	3.40 (0.31-37.55)	1.06 (0.89-12.74)
**Frequency of reading newspaper**		
Not at all	Reference (1.0)	Reference (1.0)
Less than once a week	1.68*** (1.42-1.99)	1.30** (1.06-1.58)
At least once a week	2.15*** (1.64-2.83)	1.13 (0.82-1.56)

**Frequency of listening radio**		
Not at all	Reference (1.0)	Reference (1.0)
Less than once a week	1.25*** (1.07-1.44)	1.16 (0.99-1.36)
At least once a week	1.42*** (1.23-1.64)	1.19* (1.02-1.39)
**Frequency of watching TV**		
Not at all	0.93 (0.81-1.06)	1.04 (0.87-1.23)
Less than once a week	0.85* (0.74-0.97)	0.97 (0.84-1.12)
At least once a week	Reference (1.0)	Reference (1.0)
**Wealth index**		
Poorest	Reference (1.0)	Reference (1.0)
Poorer	0.85 (0.72-1.01)	0.93 (0.77-1.12)
Middle	0.99 (0.84-1.16)	1.05 (0.861.29)
Richer	0.95 (0.81-1.13)	0.98 (0.76-1.26)
Richest	1.46*** (1.25-1.70)	1.22 (0.93-1.61)
**Level of education**		
No education	Reference (1.0)	Reference (1.0)
Primary	1.09 (0.93-1.28)	1.35*** (1.14-1.59)
Secondary	0.95 (0.84-1.08)	1.38*** (1.17-1.62)
Higher	3.27*** (2.70-3.96)	3.54*** (2.74-4.59)
**Sexual activity**		
Never had sex	0.53*** (0.46-0.61)	1.03 (0.74-1.43)
Ever had sex	Reference (1.0)	Reference (1.0)
**Ever terminated a pregnancy**		
No	Reference (1.0)	Reference (1.0)
Yes	1.58*** (1.39-1.79)	1.21*** (1.06-1.40)
**Region**		
Banjul	Reference (1.0)	Reference (1.0)
Kanifing	0.93 (0.74-1.17)	0.96 (0.75-1.21)
Brikama	0.83 (0.68-1.04)	0.95 (0.75-1.20)
Mansakonko	1.12 (0.88-1.432)	1.60** (1.17-2.18)
Kerewan	0.65*** (0.51-0.83)	0.95 (0.70-1.30)
Juntaur	1.12 (0.89-1.41)	1.92*** (1.39-2.65)
Janjanbureh	0.55*** (0.42-0.72)	0.85 (0.60-1.20)
Basse	0.69** (0.55-0.87)	1.13 (0.83-1.54)
**Community literacy level**		

Low	Reference (1.0)	Reference (1.0)
Medium	1.05 (1.10-1.43)	1.13 (0.95-1.35)
High	1.26*** (1.10-1.43)	1.12 (0.88-1.44)
**Community poverty level**		
Low	Reference (1.0)	Reference (1.0)
Moderate	0.88 (0.74-1.05)	0.90 (0.73-1.12)
High	1.29*** (1.15-1.45)	1.17 (0.91-1.51)

{***} {**} {*} shows 1%, 5% and 10% significance respectively

AOR: Adjusted odds ratio

COR: Crude odds ratio

Model I: Bivariate analysis between fistula awareness and each explanatory variable

Model II: Multivariate analysis between fistula awareness and the explanatory variables

Figures

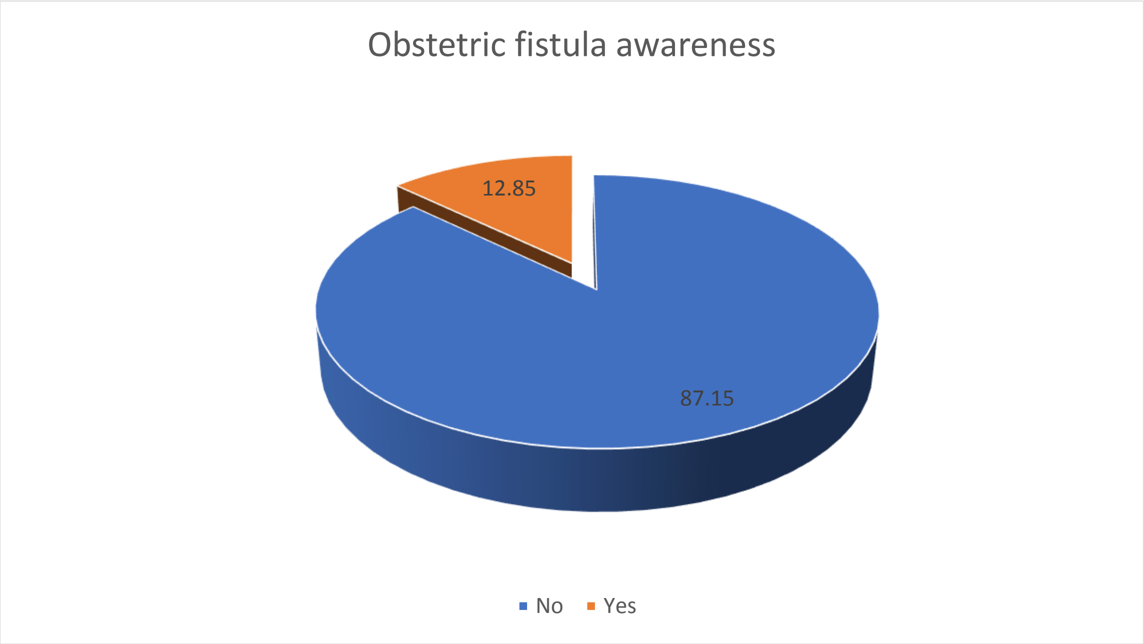

Figure1: *Prevalence of fistula awareness among women in Gambia*

Click here to access/download
Supporting Information - Compressed/ZIP File Archive
Supporting documents.zip
